# Predictors of defaulting from completion of child immunization in south Ethiopia, May 2008 – A case control study

**DOI:** 10.1186/1471-2458-9-150

**Published:** 2009-05-22

**Authors:** Henoke Tadesse, Amare Deribew, Mirkuzie Woldie

**Affiliations:** 1 Dilla University, School of Public Health, Dilla, Ethiopia; 2 Jimma University, Public Health Faculty, Department of Epidemiology and Biostatistics, P.O. Box, 205, Jimma, Ethiopia; 3 Jimma University, Public Health Faculty, Department of health service planning and management, Jimma, Ethiopia

## Abstract

**Background:**

Epidemiological investigations of recent outbreaks of vaccine preventable diseases have indicated that incomplete immunization was the major reason for the outbreaks. In Ethiopia, full immunization rate is low and reasons for defaulting from immunization are not studied well. The objective of the study was to identify the predictors of defaulting from completion of child immunization among children between ages 9–23 months in Wonago district, South Ethiopia.

**Methods:**

Unmatched case control study was conducted in eight Kebeles (lowest administrative unit) of Wonago district in south Ethiopia. Census was done to identify all cases and controls. A total of 266 samples (133 cases and 133 controls) were selected by simple random sampling technique. Cases were children in the age group of 9 to 23 months who did not complete the recommended immunization schedule. Pre-tested structured questionnaire were used for data collection. Data was analyzed using SPSS 15.0 statistical software.

**Results:**

Four hundred eighteen (41.7%) of the children were fully vaccinated and four hundred twelve (41.2%) of the children were partially vaccinated. The BCG: measles defaulter rate was 76.2%. Knowledge of the mothers about child immunization, monthly family income, postponing child immunization and perceived health institution support were the best predictors of defaulting from completion of child immunization.

**Conclusion:**

Mothers should be educated about the benefits of vaccination and the timely administration of vaccines.

## Background

Worldwide about 29,000 children under the age of five die every day, mainly from preventable causes. An Ethiopian child is 30 times more likely to die by his or her fifth birthday than a child in Western Europe [1]. Some of the deaths occur from illnesses like measles, malaria or tetanus. According to the demographic health survey (DHS, 2005) of Ethiopia, child mortality rate of the country was 132 per 1000 live births, which is one of the highest in the world. The child mortality rate in south Ethiopia was 142 per 1000 live births [2,3]. Immunization offered the greatest benefits for health, well being and survival of children than any other interventions [4]. From 1960–2002, a fifty percent reduction in under-five mortality was observed. Immunization programmes has saved the lives of nearly 4 million children [1-3]. Study showed that the cost to treat a vaccine preventable disease is 30 times more than the cost of the vaccine [5].

Epidemiological investigations of recent outbreaks of vaccine preventable diseases indicated that incomplete immunization was the major reason for the outbreaks. Moreover, a low immunization rate was the major reasons for many of the outbreaks of infectious diseases in the past two decades [6].

In 1980, the Ministry of Health of Ethiopia initiated the Expanded Program on Immunization (EPI). The objective of EPI in Ethiopia was to fully vaccinate 90% under-one children by the year 1990. The vaccination schedule of Ethiopia is based on the e recommendation of World Health Organization (WHO) for developing countries [7,8]. However, different literatures revealed that EPI schedule in Ethiopia is not completed as planned and full immunization rate is low (49.9%). As a result many children in Ethiopia do not get the benefits of immunization [9,10]. To increase the immunization coverage in Ethiopia, predictors of defaulting has to be identified through community based studies. Such community based studies are not available in Ethiopia. The objective of this study was to identify the predictors of defaulting from immunization among children between 9–23 months of age in Wonago district, south Ethiopia.

## Methods

### Study area

This unmatched case control study was conducted from February 15 to March 15, 2008 in Wonago district in south Ethiopia. Wonago is one of the densely populated districts in Ethiopia. The District has 17 rural and one urban *Kebeles *(small administrative units). The *Kebeles *in the district were stratified into urban and rural settings. Seven rural Kebeles were randomly selected by lottery method. The only urban *Kebele, namely *Wonago was also included in the study. The total population of the study Kebeles was 39600.

### Participants

The study population were children in the age group of 9 to 23 months in the selected Kebeles. Cases were children in the age group of 9 to 23 months who did not complete the recommended schedule of immunization. Controls were defined as children who were in the age group 9 to 23 months and completed the recommended schedules of immunization which include Bacillus Calmette Guerin (BCG), pentavalent, polio and measles vaccines. Children who never got vaccination were excluded in the study. Sample size was calculated using EPI-Info 6.04 statistical software (Center for Disease Control and Prevention, Atlanta, 2005). The assumptions for the sample size calculation were: proportion of illiterate mothers or caretakers of 82% among the cases and 94.4% among controls [11], 80% power, 95% confidence interval, 10% non-response rate and a case: control ratio of 1:1. The total sample size was 266 (133 cases and 133 controls). Among many exposure variables, educational status was selected since it gave maximum sample size as compared to other variables. In the selected Kebeles, first census was done to identify all cases and controls. The total sample size was distributed to each Kebele based on proportional to size allocation (Table 1). Using the sampling frame of cases and controls from the census, 133 cases and 133 controls were selected by simple random sampling technique.

**Table 1:** Distribution of the sample size in the study Kebeles in Wonago District, South Ethiopia, May, 2008

Kebele	Total Population	Total under-five children	Total number of source population(age 9 to 23 months)		Sample size in each Kebele		
			
			Source population for Controls (fully vaccinated children)	Source population for cases(Defaulters)	Controls	cases	total
Wonago	4912	480	61	36	19	12	31
Tokicha	4849	463	29	32	9	10	19
Bale Busika	4895	493	40	77	13	25	38
Jemjemo	4729	443	53	43	17	14	31
Dodoro	5699	590	109	42	35	14	49
Hase Haro	4849	473	16	40	5	13	18
Dako	4918	501	39	90	12	29	41
Kara Soditti	4749	451	71	52	23	17	39

Total	39600	3894	418	412	133	133	266

### Measurements

The data was collected by trained nurses using Amharic version structured questionnaire. The structured questionnaire was adopted from world health organization (WHO) and Demographic health survey of Ethiopia (DHS) [2]. The content of the questionnaire included: socio-demographic variables, questions related to maternal and child health services, knowledge of the mothers about vaccination, perception of mothers about health institution support and immunization status of children. Prior to data collection, the questionnaire was pre-tested on 5% of the sample on similar population. The reliability of the items in the questionnaires was tested using Cronbach's alpha.

### Data Processing and analysis

The collected data were entered into computer and analyzed using SPSS version 15.0 statistical software. The data was cleaned for inconsistencies and missing values. Different predictors of defaulting from immunization were assessed using logistic regression model. In the first model, the effects of socio-demographic variables were assessed. The effect of maternal and child health services were assessed in the second model. In the third and fourth model, perception and knowledge related variables were assessed. From all the models, variables which had significant association with dependent variable (P < 0.05) were entered to the final regression model using stepwise logistic regression technique.

### Operational definitions

The following operational definitions were used:

#### Defaulter

If the child missed at least one of the recommended vaccines, he/she was considered as defaulter.

#### Complete immunization

Complete immunization was considered if the child took all the recommended vaccines including BCG, pentavalent, polio and measles vaccine by the age of 23 months.

#### Perception

Perceived health institution support, perception on the presence barrier of immunization, and perception about immunization were assessed using Likert Scale questions. Mean score for each constructs was computed and dichotomized into positive and negative. If respondents scored below the mean, he/she would be labeled as having negative perception.

#### Dropout rate (DOR)

The rate difference between the initial vaccine (BCG or Pentavalent I) and the final vaccines (Pentavalent III or Measles).





### Ethical consideration

Ethical clearance was obtained from the Ethical review Committee of Jimma University. Before data collection, written consent was obtained from the respondents.

## Results

From the total 266 sampled children, 264 children (132 cases and 132 controls) were included in the study with response rate of 99.2%. The mean age of the children was 18.66 months with standard deviation (SD) of 4.46. Majority of the children (82%) were in the age group 13–23 months. Males accounted 51.5% of the total children.

None of the mothers did know newly added vaccines, namely the vaccines against hepatitis B and Haemophilus influenza type B. Majority of mothers and immediate caretakers (78.8%) had positive attitude toward health institution support. Most of the respondents (76%) believed that immunization was beneficial for their children in preventing the occurrence and spread of diseases. From the census, we identified that 418 (41.7%) of children had taken all the recommended immunization. Whereas 412 (41.5%) and 171 (17%) of the children were partially immunized and never vaccinated respectively. The evidences of vaccination for majority of children (77%) was interview and only 60(22.7%) of the children had vaccination card. Similarly, the majority of evidences of vaccination (86.4%) among the cases were history. The vaccination rate varied among series of vaccines. The BCG vaccination rate was 87% in the total children (cases and controls) and 73.5% among the cases. Similarly, measles vaccination rate was 58.7% in total children (both cases and controls) & 17% among the cases. The BCG: measles dropout rate for total children was 32.3% and the dropout rate among the cases was 76.2%.

Of the total mothers/caretakers, 108 (40.9%) took their children to health institution for health and health related services other than immunization. Most of the mothers and immediate caretakers 95 (87.2%) took their children to health institution to seek help for some kind of illnesses. The rest of them took their children for growth monitoring and follow up. However; only 54.1% respondents were advised or informed about vaccination during their visit. The missed opportunity rate was 46.3%.

### Socio-demographic predictors of defaulting

Of the socio demographic characteristics of the respondents, only monthly family income was found to be predictor of defaulting. The other socio-demographic variables such as family size, age of the mother or immediate care taker, occupational status, ethnicity, religion, parity, and educational status were not associated with of defaulting. Monthly family income had retained its significance after adjusting other socio-demographic characteristics. Mothers or immediate caretakers who had monthly family income of 44–88 USD were 81.1% less likely to have defaulter children than mothers or immediate caretakers who had monthly family income below < 22 USD, [OR = 0.430 (95% CI: 0.20, 0.94)].

### The effect of Maternal and child health services on defaulting

There was a significant association between utilization of postnatal care (PNC) service and completion of child immunization **(p-value < 0.05**). Mothers who did not use PNC service after delivery of the child under study were 6 times more likely to have defaulter children than mothers who did use PNC services, [OR = 5.8 (95% **CI: 1.68, 27.29**)]. Mothers who did not postpone vaccination schedule were 98% times less likely to have defaulter children as compared with mothers who ever postponed vaccination schedule (Table 2).

**Table 2:** Health services factors and defaulting from completion of child immunization in Wonago district, South Ethiopia, May 2008

***Variable***	***Cases***	***Control***	***Crude OR (95%CI)***	***Adjusted OR (95%CI)***
**Postponed vaccine Schedule**				
Yes	110(94.8%)	6(5.2%)	1	1
No	22(14.9%)	126(85.1%)	0.01 (0.004, 0.024)	0.02 (0.002, 0.264)
**Ever ANC visit**				
Yes	64(41.6%)	90(58.4%)	1	1
No	65(65%)	35(35%)	2.61(1.551,4.398)	1.01 (0.117, 8.721)
**Current PNC visit**				
Yes	13(35.1%)	24(64.9%)	1	1
No	9(75%)	3(25%)	5.54(1.273,24.14)	19.52(1.685, 226.29)
**Evidence of vaccination**				
Vaccination card	18(30%)	42(70%)	1	1
History	114(55.9%)	90(44.1%)	2.96(1.594,5.480)	1.79(0.241, 13.233)

### Perception of mothers as predictors of defaulting

Perception of mothers/immediate caretakers of the children toward health institutions support had significant association with completion of child vaccination (p-value < 0.001). Respondents who had negative perception toward health institutions support were 2.7 more likely to have defaulter children as compared to their counterparts, [OR = 2.71,(95% CI: 1.39, 5.26)] (Table 3).

**Table 3:** The effect of perception of mothers/caretakers on defaulting from completion of child immunization in Wonago District, South Ethiopia, May 2008.

***Variables***	***Cases***	***Control***	***Crude OR (95%CI)***	***Adjusted OR (95%CI)***
**Perceived health institution support**				
Positive attitude	93 (44.7%)	115(55.3%)	1	1
Negative attitude	39 (69.6%)	17(30.4%)	2.83(1.51, 5.35)	2.71(1.39,5.26)
**Perception about immunization**				
Positive	35(57.4%)	26(42.6%)	1	1
Negative	97(47.8%)	106(52.2%)	1.47(0.382, 1.44)	1.18(0.635, 2.18)
**Perception on presence of barriers of immunization**				
positive	27(57.4%)	20(42.6%)	1	1
Negative	105(48.4%)	112(51.6%)	1.44 (0.762, 2.725)	1.03 (0.513, 2.058)

### Knowledge as predictors of defaulting

Knowledge of mothers or immediate caretakers about schedule of vaccines had significant association with completion of immunization **(p-value < 0.001**). Mothers who did know the schedules of vaccine were 3 times more likely to vaccinate their children fully than mother who didn't know vaccine schedule, [OR = 3 (95% **CI: 1.4, 6.3**)]. Mothers or immediate care taker who did not know the benefits of immunization in preventing the occurrence of epidemic were 6.4 times more likely to have defaulter children than mother who knew the benefits, [OR = 6.4, (95% **CI: 0.43, 9.53**)]. Knowledge about measles and polio vaccines were also significantly associated with completion of child immunization after controlling the effect of other variables (Table 4).

**Table 4:** Immunization related Knowledge and defaulting from completion of child immunization in Wonago district, South Ethiopia, May 2008

***Variable***	***Cases***	***Control***	***Crude OR (95%CI)***	***Adjusted OR (95%CI)***
**Know BCG**				
Yes	60(40.8%)	87(59.2%)	1	1
No	29(60.4%)	19(39.6%)	2.21(1.14,4.31)	0.89 (0.38, 2.15)
**Know newly added vaccine**				
Yes	56(40%)	84(60%)	1	1
No	33(60%)	22(40%)	2.250(1.190,4.253)	0.60 (0.26, 1.46)
**Know measles**				
Yes	6(7.9%)	70(92.1%)	1	1
No	83(69.7%)	36(30.7%)	26.89 (10.7,67.56)	34.72 (12.74, 94.64)
**Knowledge on schedule of vaccines**				
Yes	68(41.5%)	96(58.5%)	1	1
No	21(67.7%)	10(32.3%)	2.965 (1.313,6.69)	3.01(1.42, 6.35)
**Knowledge on schedule of Polio Vaccine**				
Yes	35(36.5%)	61(63.5%)	1	1
No	97(57.7%)	71(42.3%)	2.381(1.421,3.990)	6.52(1.35,31.39)
**Knowledge on the benefit of vaccine**				
Yes	8(26.7%)	22(73.3%)	1	1
No	78(48.4%)	83(51.6%)	2.584(1.087,6.145)	6.359(0.428,94.543)

### Independent predictors of defaulting

In the final logistic regression model, we have identified that monthly family income, postponing of child immunization schedule, perceived health institute support, knowledge about measles and benefits of vaccination were the independent predictors. Mothers who had poor knowledge about the benefit of vaccines were 6 times more likely to have defaulter children than mothers who had good knowledge, [OR = 6.3, (95%CI: 1.24, 9.53)]. Similarly, mothers who had negative perception towards health institution support were 2.3 times more likely to have defaulter children than mothers with positive attitude, OR = 2.3 [95%CI: 0.67, 7.6](Table 5).

**Table 5:** Independent predictors of defaulting from completion of child immunization in Wonago district, South Ethiopia, May 2008

***Variable***	***Cases***	***Controls***	***Crude OR (95%CI)***	***Adjusted OR (95%CI)***
**Monthly family income in USD**				
< 22	117(89.3%)	96(72.7%)	1	1
22–44	11(8.4%)	21(15.9%)	0.43(0.197, 0.936)	2.79(0.550,14.122)
45–88	3(2.3%)	15(11.4%)	0.16(0.046,0.584)	0.32(0.053,0.937)
**Postponed vaccines Schedule**				
Yes	22(14.9%)	126(85.1%)	1	1
No	110(94.8%)	6(5.2%)	0.010(0.004,0.024)	0.002(0.000,0.025)
**Perceived health institution support**				
Positive	93 (44.7%)	115(55.3%)	1	1
Negative	39 (69.6%)	17(30.4%)	2.83(1.508,5.35)	2.32(1.52, 7.63)
**Know measles**				
Yes	6(7.9%)	70(92.1%)	1	1
No	83(69.7%)	36(30.7%)	26.89 (10.7,67.56)	84.89(8.220,876.883)
**Knowledge on the benefit of vaccine**				
Yes	8(26.7%)	22(73.3%)	1	1
No	78(48.4%)	83(51.6%)	2.58(1.087,6.145)	6.36(1.24, 9.53)
**Evidence of vaccination**				
Vaccination card	18(30%)	42(70%)	1	1
History	114(55.9%)	90(44.1%)	2.96(1.594,5.480)	1.79(0.241, 13.233)

## Discussion

All the study Kebeles had access to immunization services monthly at outreach and weekly at health institutions. However, significant proportion of children (42%) didn't complete the recommended immunization schedule. The dropout rate in our study was higher as compared to a study conducted in Kenya [12] which was 22.6%. However, it is lower than that of the national EPI cluster survey result (35.6%) [13]. Determinants of receipt of immunization are complex and interwoven. This study identified several socio-behavioral factors which affected child immunization. The most important variables that predicted defaulting from immunization were perceived health institution support, monthly family income, postponing child immunization schedule, and knowledge of the mothers/caretakers about the benefit of immunization. Of the socio demographic characteristics of the respondents, only monthly family income was found to be predictor of defaulting from completion of child immunization in this study. A similar study by Renstein showed that income had consistently affected receipts of immunization [14]. Other socio-demographic variables were not associated with defaulting. Mothers or immediate caretakers who had negative attitude about health institution support were 2.3 times more likely to have defaulter children than mothers or immediate caretakers who had positive attitude. Similar finding was obtained from other study which showed that the barriers of completion of child immunization were poor knowledge, attitude and perception of health institution support [15]. This study had identified that maternal knowledge about immunization was one of the major reasons for defaulting. Other literatures had similar findings elsewhere [16,17]. It was shown that postponing child immunization schedule was a predictor of completion of child immunization. Similarly, a facility based study conducted in Nigeria showed that the most common reason given by respondents for defaulting of child immunization were illnesses of children and postponing immunization [18].

We tried to assess many predictors of defaulting from child immunization using a community based case control study. We had identified that knowledge and perception of the mothers had contributed for defaulting from child immunization. However, our study didn't address the effect of lack of access to health care and shortage of trained human resources as predictors of defaulting from child immunization. Lack of infrastructure and adequate human resources might be the root causes of defaulting from child immunization. Our study had also some limitations which included recall bias where mother might forget the immunization status of their children and misclassification. Due to forgetfulness of the mothers/caretakers about the status of immunization of their children, cases and controls could be misclassified.

## Conclusion

It is concluded that behavioral factors like knowledge, attitude and perception of mother had strong association with defaulting from child immunization. Monthly family income and postponing of child immunization schedule were also the other independent predictors of defaulting. Mothers should be educated about the benefits of vaccination and the timely administration of vaccines.

## Competing interests

The authors declare that they have no competing interests.

## Authors' contributions

**HT **was involved in the conception, design, analysis and report writing. **AD **had been involved in the design, analysis and interpretation of the data, report writing and dissemination of the article. **MW **assisted with the conception and designing the study and critically reviewed the manuscript.

## Pre-publication history

The pre-publication history for this paper can be accessed here:


